# Fibrin deposition on bovine pericardium tissue used for bioprosthetic heart valve drives its calcification

**DOI:** 10.3389/fcvm.2023.1198020

**Published:** 2023-07-31

**Authors:** Bastien Poitier, Jeanne Rancic, Ulysse Richez, Julie Piquet, Salma El Batti, David M. Smadja

**Affiliations:** ^1^ Université de Paris Cité, Innovative Therapies in Haemostasis, INSERM UMR-S1140, Paris, France; ^2^ Cardiovascular Surgery Department, AP-HP, Georges Pompidou European Hospital, Paris, France; ^3^ Biosurgical Research Lab (Carpentier Foundation, Université de Paris Cité and AP-HP), Paris, France; ^4^ Hematology Department, AP-HP, Georges Pompidou European Hospital, Paris, France

**Keywords:** bioprosthetic heart valve, pericardium tissue, thrombosis, fibrin, calcification

## Abstract

**Background:**

Bioprosthetic heart valves (BHVs) are less thrombogenic than mechanical prostheses; however, BHV thrombosis has been proposed as a risk factor for premature BHV degeneration.

**Objectives:**

We aimed to explore whether fibrin deposition on bovine pericardium tissue could lead to calcification.

**Method:**

Fibrin clot was obtained by blending three reagents, namely, CRYOcheck™ Pooled Normal Plasma (4/6), tissue factor + phospholipids (Thrombinoscope BV), and 100 mM calcium (1/6), and deposited on pericardium discs. Non-treated and fibrin-treated bovine pericardium discs were inserted into the subcutaneous tissue of 12-day-old Wistar rats and sequentially explanted on days 5, 10, and 15. Calcium content was measured with acetylene flame atomic absorption spectrophotometry. Histological analysis was performed using hematoxylin–eosin staining, Von Kossa staining, and immunohistochemistry.

**Results:**

Calcification levels were significantly higher in fibrin-treated bovine pericardium discs compared to those in non-treated bovine pericardium discs (27.45 ± 23.05 µg/mg vs. 6.34 ± 6.03 µg/mg on day 5, 64.34 ± 27.12 µg/mg vs. 34.21 ± 19.11 µg/mg on day 10, and 64.34 ± 27.12 µg/mg vs. 35.65 ± 17.84 µg/mg on day 15; *p* < 0.001). Von Kossa staining confirmed this finding. In hematoxylin–eosin staining, the bovine pericardium discs were more extensively and deeply colonized by inflammatory-like cells, particularly T lymphocytes (CD3^+^ cells), when pretreated with fibrin.

**Conclusion:**

Fibrin deposition on bovine pericardium tissue treated with glutaraldehyde, used for BHV, led to increased calcification in a rat model. BHV thrombosis could be one of the triggers for calcification and BHV deterioration.

## 1. Introduction

Valvular heart disease is a very common pathology that affects patients worldwide. Its prevalence is rising with the aging of the population, as degenerative valve disease is the most common etiology, in particular, in industrialized countries (1). Heart valve replacement is increasingly performed with bioprosthetic heart valves (BHVs) that allow good hemocompatibility without the lifelong use of anticoagulation treatment but raise the issue of durability (2). Indeed, mechanical prostheses have long durability but are exposed to thromboembolic complications or hemorrhagic risk due to anticoagulation treatment (3). BHVs are fabricated mostly from bovine pericardium treated with glutaraldehyde and undergo structural valve deterioration (SVD) over time. It remains an unsolved problem that glutaraldehyde-treated bovine pericardium is subject to degenerative processes involving rigidity and calcification. Toxicity of glutaraldehyde has been proposed even if its involvement in long-term degenerative processes is unclear (4, 5). Recently, we described that glutaraldehyde-treated bovine pericardium inside a bioprosthetic artificial heart was quickly recovered by fibrin and newly formed endothelial cells (6, 7). Although explanted BHVs are infiltrated by inflammatory cells but also fibrotic and endothelial cells (8, 9), their involvement in SVD is always a question of debate.

Although BHVs are less thrombogenic than mechanical prostheses, BHV thrombosis is an increasingly recognized entity that raises the issue of initial anticoagulation treatment in this population. Reduced leaflet motion because of thrombosis or impaired leaflet coaptation has been described, which may or may not be associated with thromboembolic complications (10, 11). Petrescu et al. (12) found that at 10 years after BHV thrombosis, the probability of needing valve re-replacement was 70% in patients with BHV thrombosis vs. 31% in matched control subjects (*p* < 0.001). The causal relationship between BHV thrombosis and SVD is a key feature to explore.

This study aimed to explore the role of fibrin in bovine pericardium calcification. Furthermore, we compared the calcium content of pericardium tissue with and without *in vitro* fibrin recovery followed by subcutaneous implantation in rats.

## 2. Methods

### 2.1. Tissue preparation

**Figure 1:**
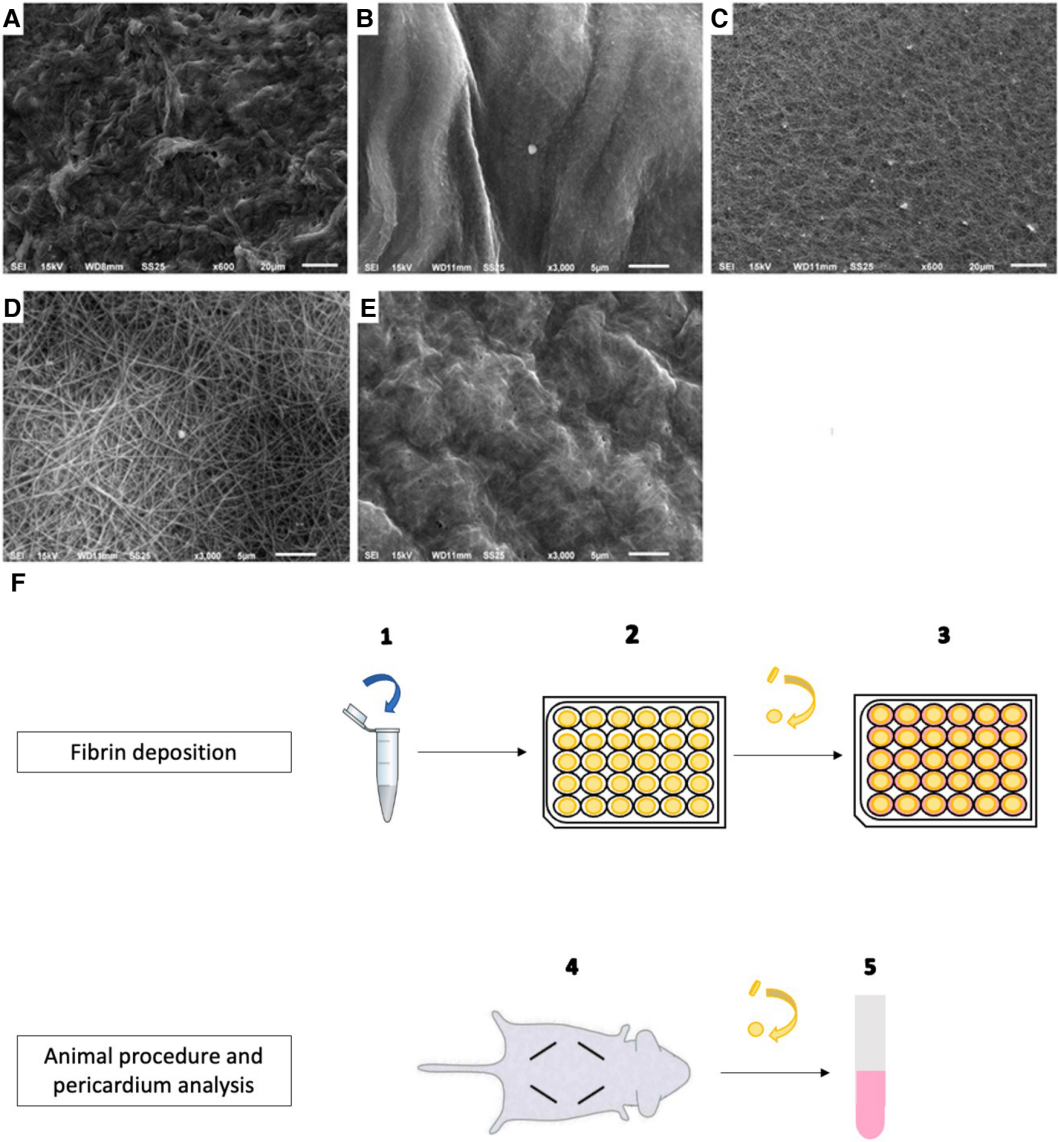
Fibrin deposition on bovine pericardium: preparation, implantation, and scanning electron microscopy analysis. (**A**) Non-treated pericardium tissue surface (magnification = ×600; scale bars = 20 μm). (**B**) Fibrin-treated pericardium tissue surface with 10 µl of fibrin solution (magnification = ×3,000; scale bars = 5 μm). (**C**) Fibrin-treated pericardium tissue surface with 20 µl of fibrin solution (magnification = ×600; scale bars = 20 μm). (**D**) Fibrin-treated pericardium tissue surface with 20 µl of fibrin solution (magnification = ×3,000; scale bars = 5 μm). (**E**) fibrin-treated pericardium tissue surface with 50 µl of fibrin solution (magnification = ×3,000; scale bars = 5 μm). (**F**) Protocol for pericardium tissue preparation and implantation, after washing.

Neovasc® pericardium tissue was stored in 0.6% glutaraldehyde and was cut into discs of 0.8 cm in diameter. The discs were washed in cold tris-buffered saline (TBS) + fetal bovine serum (FBS) 10% for 5 days at 4°C with gentle shaking (2 discs/ml) for flushing glutaraldehyde. Three baths were realized on the first day, and then rinsing baths were changed three times during the 5 days. Then, two groups were prepared: pericardium discs not treated with fibrin and pericardium discs treated with fibrin on both faces. The fibrin recoveries were obtained by blending three reagents as follows: tissue factor + phospholipids (Thrombinoscope BV) (1/6 of the total volume) and CRYOcheck™ Pooled Normal Plasma (4/6 of the total volume) were mixed in an Eppendorf. At the last moment, 100 mM of Ca (1/6 of the total volume) was added. Three volumes of this final solution were tested for deposition on the pericardium disc before planning animal implantation: 10, 20, and 50 µl. Thus, immediately after adding 100 mM of Ca, 10, 20, or 50 µl of this fibrin solution was disposed of on each pericardium disc placed beforehand in a 24-well plate. The discs were turned over after 2 h incubation at 37°C, and the same operation of fibrin clot deposition was repeated on the other side. After 2 h of re-incubation, 1 ml of EGM2-FBS 10% was added to each well, and the plate was incubated for 24 h (Figure 1).

### 2.2. Scanning electron microscopy (SEM)

Before initiating the animal procedure, we explored on SEM the surface aspect of the pericardium tissue with fibrin recovery. The samples were fixed with 0.6% glutaraldehyde and then post-fixed with 1% osmium tetroxide. Dehydration with ethanol and drying with hexamethyldisilazane were performed before the samples were mounted on a stub and metalized.

### 2.3. Surgical procedure

Animal implantation was scheduled for the next day. Twelve-day-old Wistar rats (Janvier Labs, Le Genest St. Iles, France) were used for the experimentation as previously described (4, 13). Sedation was performed with 2.5% isoflurane, and analgesia was allowed by subcutaneous injection of buprenorphine (0.05 mg/kg). The skin was cleaned with betadine. Four subcutaneous incisions in the dorsal wall of the rats were performed, and discs were placed (four discs/rat). Incisions were closed by skin clips. The pericardium discs were explanted after 5, 10, and 15 days. The rats were euthanized by an intraperitoneal injection of pentobarbital (Euthasol Vet 0.35 ml/kg) after sedation. The discs were rinsed with 0.09% saline solution and stored at −20°C before analysis. The samples allocated for histology were fixed in 4% paraformaldehyde.

### 2.4. Disc analysis

#### 2.4.1. Calcium dosage

The first part of the discs was allocated to calcification quantification. The discs were hydrolyzed in a 68% nitric acid bath at 90°C for 20 min, allowing calcium dosage, performed by air–acetylene flame atomic absorption spectrophotometry (VARIAN AA240). The results are given in µg/mg of pericardium.

#### 2.4.2. Histology

The second part of the discs was allocated to histology. The samples were dehydrated with ethanol and paraffin-embedded. Hematoxylin–eosin staining (H&E) and Von Kossa staining were then carried out to examine cell morphology and calcium deposit, respectively. Immunohistochemistry (IHC) with anti-CD3 [monoclonal antibody (SP7), ab16669, Abcam] was performed to highlight T-cell infiltration in explanted pericardium discs. T-cell infiltration was quantified using ImageJ software on 10 different slides. Slide staining was observed with an optical microscope.

### 2.5. Statistical analyses

A Mann–Whitney *U* test was performed to compare calcium dosage at any time and for CD3^+^ cell quantification on IHC staining. All analyses were two-sided, and *p* < 0.05 was considered statistically significant. Statistical analyses and curves were realized using GraphPad software.

## 3. Results

### 3.1. Fibrin pre-treatment of bovine pericardium significantly increased calcium content after rat implantation

**Figure 2:**
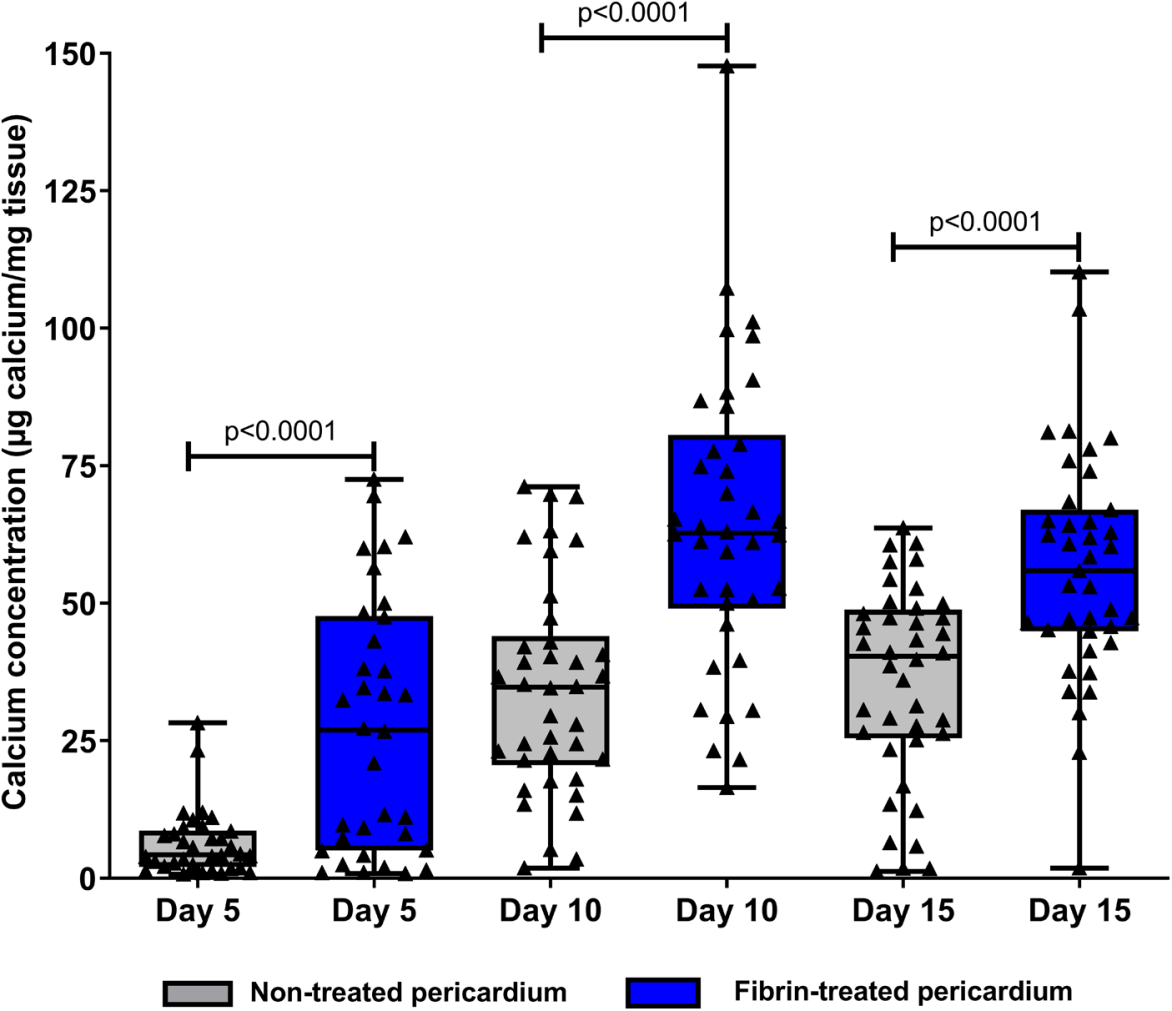
Dosage of tissue calcium levels between fibrin-treated and non-treated bovine pericardium tissues after subcutaneous implantation in rats for 15 days.

To test the ability of fibrin in modifying the calcification of pericardium discs, fibrin clots were prepared in both faces of the pericardium *in vitro* as detailed in Figure 1 and then observed by scanning electron microscopy. As observed in Figure 1, while 20 µl of fibrin solution was used, a continuous sheet was formed that was underlaid with a framework of barely apparent polymerized fibrin structuring a real network, as previously described by Amelot et al. (14). In addition, a deposition of 10 µl of the fibrin solution did not allow us to obtain a visible mesh network, and a deposition of 50 µl led to a too dense network. Therefore, we determined that a deposition of 20 µl of our fibrin solution was the most relevant, and we used it for the animal implantation. Then, a total of 60 rats were operated. A total of 120 non-treated pericardium discs and 120 fibrin-treated pericardium discs were analyzed. The macroscopic analysis did not show any local inflammatory reaction for any rat. In addition, 108 discs from each group were allocated to the calcification assay. We found that pericardium tissue calcification significantly increased between 5 and 10 days, but did not show further progression beyond this point (*p* = 0.45 between days 10 and 15 for non-treated pericardium discs and *p* = 0.17 between days 10 and 15 for fibrin-treated pericardium discs) (Figure 2). Moreover, when the pericardium discs were treated with fibrin, we observed a significant increase in calcification levels (6.34 ± 6.03 µg/mg vs. 27.45 ± 23.05 µg/mg, *p* < 0.001 at day 5; 34.21 ± 19.11 µg/mg vs. 64.34 ± 27.12 µg/mg, *p* < 0.001 at day 10; 35.65 ± 17.84 µg/mg vs. 56.30 ± 20.77 µg/mg, *p* < 0.001 at day 15, respectively, for non-treated pericardium discs and fibrin-treated pericardium discs; Figure 2).

### 3.2. Fibrin pre-treatment of bovine pericardium promotes inflammatory-like cell infiltration after subcutaneous implantation in rats

**Figure 3:**
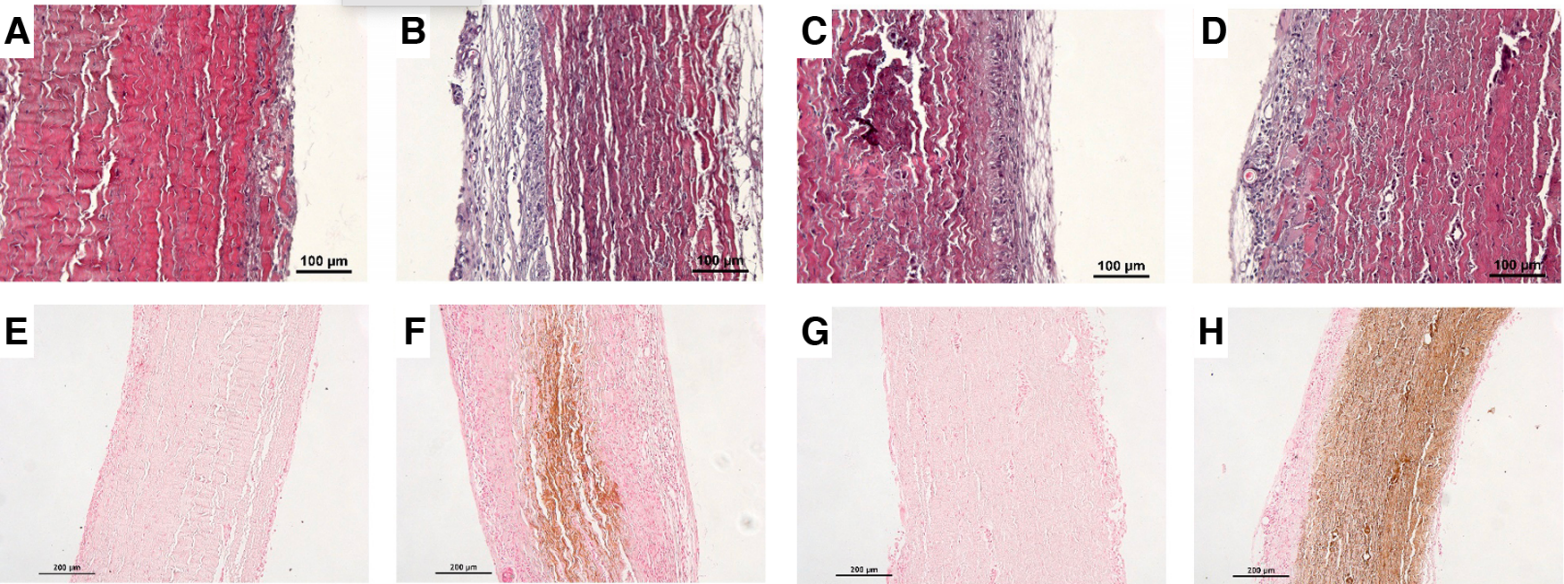
Panel of histological analyses. Hematoxylin–eosin staining for (**A**) non-treated pericardium tissue at day 5, (**B**) non-treated pericardium tissue at day 15, (**C**) fibrin-treated pericardium tissue at day 5, and (**D**) fibrin-treated pericardium tissue at day 15. Von Kossa staining for (**E**) non-treated pericardium tissue at day 5, (**F**) non-treated pericardium tissue at day 15, (**G**) fibrin-treated pericardium tissue at day 5, and (**H**) fibrin-treated pericardium tissue at day 15.

**Figure 4:**
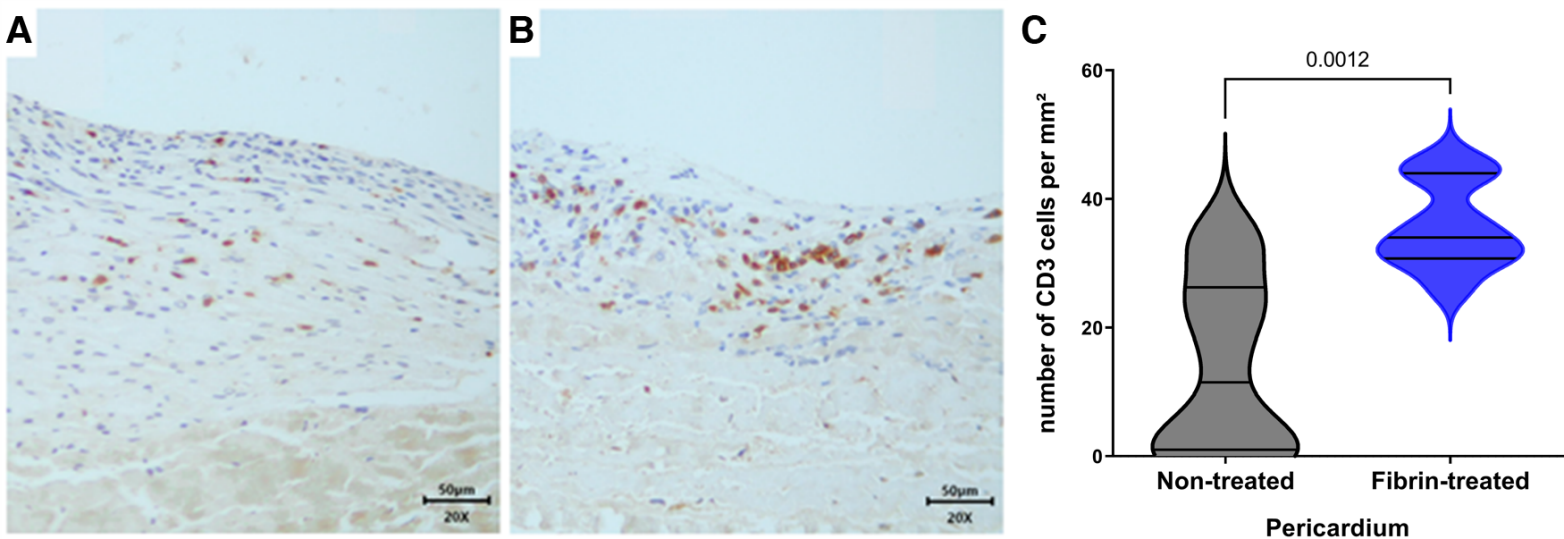
IHC analysis with anti-CD3. (**A**) Non-treated pericardium tissue at day 15 (magnification = ×20; scale bars = 50 μm). (**B**) Fibrin-treated pericardium tissue at day 15 (magnification = ×20; scale bars = 50 μm). (**C**) Quantification of CD3^+^ cells in implanted pericardium treated or not treated with fibrin.

We found on H&E staining that the non-treated pericardium discs were colonized by inflammatory-like cells only in the superficial layer on day 5. On day 15, a fibrin network appeared, and inflammatory-like cells were found to be present in deeper layers of the pericardium (Figures 3A,B). The fibrin-treated pericardium discs were largely colonized by inflammatory-like cells from day 5, into deeper layers of the pericardium, in comparison with the non-treated pericardium discs. Moreover, neo-capillaries were observed on day 15 (Figures 3C,D). As demonstrated in Figure 3, we observed only a slight calcification area in the non-treated pericardium discs at day 15 (Figure 3F) in the Von Kossa staining, whereas the fibrin-treated pericardium discs presented a large area of calcification (brown color, Figure 3H) confirming an increased calcification potential of fibrin on top of bovine pericardium treated with glutaraldehyde. To further investigate immune infiltrate composition, we performed anti-CD3 (targeting T cells), anti-MPO (targeting neutrophils), and anti-CD68 (targeting monocytes and macrophages) labeling. Significant infiltration of monocytes and macrophages in both groups (non-treated and fibrin-treated pericardium discs) was found without any significant difference. A slight infiltration of neutrophils in both groups on day 15 was observed, with no difference observed. The CD3^+^ cell infiltration demonstrated a higher level of T lymphocytes in the fibrin-treated pericardium discs compared to that in the non-treated ones (mean: 35.8 cells/mm^2^, SD: 6.79 vs. mean: 14.1 cells/mm^2^, SD: 13.8; *p* = 0.0012) (Figure 4).

## 4. Discussion

In this study, we demonstrated here an increased calcification process in a rat model after fibrin recovery of bovine pericardium tissue treated with glutaraldehyde. To our knowledge, we provided here the first exploration *ex vivo* of the calcification potential of fibrin on top of bovine pericardium used for BHVs in clinics for the past 30 years. Fibrin-treated pericardium also showed an increased ability to be infiltrated by inflammatory cells. Indeed, inflammation and related potential residual immunogenicity could also participate to increase the calcification process as previously described (15).

Non-degenerated explanted bovine pericardium biomaterials are rarely available for in-depth studies. However, an early phase of fibrin deposit has already been described in four explanted aortic bioprostheses made of porcine pericardium (16, 17). In a closer field, we previously demonstrated the deposition of a compact fibrin network on bovine pericardium tissue inside a bioprosthetic total artificial heart (6). Moreover, we observed the incorporation of inflammatory cells and platelets in the fibrin network in all the membrane blood-contacting surfaces before starting newly formed endothelial cells on top of the pericardium tissue of the hybrid membrane (6). Unfortunately, this fibrin deposition can result in real thrombus formation. BHV thrombosis is based on the principles of Virchow's triad. First, artificial surfaces promote clot formation through plasma protein deposition from circulating blood, notably fibrinogen, fibronectin, and Von Willebrand factor, mediating platelet adhesion and then activation of the cascade coagulation (10). Hemodynamic factors, such as turbulences or low cardiac output, and hypercoagulability are also involved. Fibrin deposition and the incidence of BHV thrombosis are underestimated due to its potential subclinical presentation. The occurrence of hypoattenuated leaflet thickening on computed tomography imaging has been described as up to 30% at 1 year after transcatheter aortic valve replacement (TAVR) (18). There is a growing body of evidence linking subclinical BHV thrombosis to premature SVD. Calcification within explanted BHV leaflets has been described as colocalized predominantly with regions of thrombus formation, raising the hypothesis that BHV thrombosis might be a trigger to valve degeneration (19). Outside valve diseases, recent data proposed that fibrin deposition is at the origin of cartilage calcification (20). This is in line with our results since we demonstrated that fibrin-treated bovine pericardium discs were significantly more calcified than non-treated ones after implantation in rats.

However, the mechanism linking BHV thrombosis and premature SVD remains unclear. It has been widely reported that fibrin can induce inflammatory responses (21, 22). Furthermore, Sakaue et al. (23) suggested that the initial deposition of fibrinogen from circulating blood leading to fibrin formation results in focal accumulation of plasminogen-rich macrophages on the surface of bioprosthetic cusps. Plasminogen would trigger focal calcification by inducing an inflammatory response. It is well known that macrophages contribute directly to tissue calcification in the cardiovascular system (24, 25). Also, myocardial fibrin deposits after heart transplantation are predictive of coronary artery disease and graft rejection, indicating an association between fibrin deposition and immune responses (26).

In our study, we found that fibrin-treated bovine pericardium discs were more largely and more deeply infiltrated with inflammatory-like cells, while they were also significantly more calcified. This finding supports the hypothesis that inflammatory cells may be involved in BHV calcification. Furthermore, we showed that T-cell infiltration was higher in fibrin-treated pericardium discs. Dahm et al. (27) reported that glutaraldehyde-tanned bovine pericardium can induce lymphocytic responses when implanted in rats and guinea pigs, leading to a “host vs. graft” reaction. Thus, we can speculate that fibrin deposition could be responsible for pericardium calcification through at least in part inflammatory cell recruitment, notably T cells. We recently proposed as a key in preventing clinical BHV thrombosis a short-term and specific contact-phase inhibition on top of long-term single antiplatelet therapy (SAPT) (28) despite the absence of clear clinical benefit in particular after TAVR. However, initial anticoagulation therapy after BHV implantation remains a point of debate. The European recommendation [European Society of Cardiology (ESC)/European Association for Cardio-Thoracic Surgery (EACTS)] guidelines recommend considering low-dose aspirin or oral anticoagulation using vitamin K antagonist for the first 3 months after surgical BHV implantation (class IIa), while lifelong SAPT is recommended after TAVR but not oral anticoagulation therapy, in patients with no baseline indication for oral anticoagulation (class I) (29). The American recommendation [American College of Cardiology (ACC)/American Heart Association (AHA)] guidelines recommend also SAPT following TAVR but with class IIa. Furthermore, unlike the ESC/EACTS, the ACC/AHA guidelines suggest also either the use of dual antiplatelet therapy or vitamin K antagonist anticoagulation for the first 3 months following TAVR, with class IIb (30). Bhogal et al. (31) have demonstrated that anticoagulation therapy offers an evident protective effect on aortic valve leaflet motion, which could be a consequence of fibrin deposit inhibition. The fibrin-induced calcification process we described in the rat model needs to be confirmed in large animals. Furthermore, associations between anticoagulation regimen and fibrin deposit on BHV, but also between anticoagulation regimen and BHV longevity, particularly after TAVR, need to be more deeply explored. Organs from gene-edited pigs have been proposed for organ replacement with, in particular, overexpression of anticoagulant molecules (32). BHV from this kind of animal could be a good option to decrease fibrin deposits and potentially increase BHV longevity.

Subcutaneous implantation has certain limitations as it lacks direct contact between the pericardium tissue and circulating blood. Furthermore, the mechanisms of calcification involved in the rat model may differ from those in humans. However, the subcutaneous implantation in a 12-day-old Wistar rat model has been deemed sufficiently reliable for preclinical studies of xenograft calcification (4, 13). We suggest that this preliminary study will serve as an initial step toward more attention on fibrin deposition after BHV implantation. Further investigations in large animals, such as juvenile sheep (33, 34), could be of interest to validate our results and assess the impact of the antithrombotic treatment on pericardial calcification under conditions of circulating blood.

All in all, we demonstrate here for the first time that fibrin deposition on bovine pericardium tissue, treated with glutaraldehyde and used for BHV, led to calcification in the rat model. The initial antithrombotic treatment after BHV implantation remains a point of debate. We suggest that, beyond preventing hemodynamic risks and thromboembolic complications related to BHV thrombosis, it could also be important to prevent the risk of early SVD through the inhibition of fibrin deposits.

## Data Availability

The raw data supporting the conclusions of this article will be made available by the authors, without undue reservation.
